# Group cognitive behavioural therapy (GCBT) versus treatment as usual (TAU) in the treatment of irritable bowel syndrome (IBS): a study protocol for a randomized controlled trial

**DOI:** 10.1186/s12876-020-1157-z

**Published:** 2020-02-04

**Authors:** Shino Kikuchi, Yuki Oe, Yohei Sasaki, Hirono Ishii, Yuri Ito, Masaru Horikoshi, Takashi Sozu, Hiroshi Seno, Toshi A. Furukawa

**Affiliations:** 1 Department of Gastroenterology and Hepatology, Kyoto University Graduate School of Medicine, 54 Kawahara-cho, Shogoin, Sakyo-ku, Kyoto, Japan; 2 Department of Health Promotion and Human Behavior, Kyoto University Graduate School of Medicine / School of Public Health, Yoshida Konoe-cho, Sakyo-ku, Kyoto, Japan; 3 National Center for Cognitive Behavior Therapy and Research, National Center of Neurology and Psychiatry, 4-1-1 Ogawa Higashi, Kodaira, Tokyo Japan; 4 Department of Computer Science, Tokyo University of Science, 6-3-1 Shinjyuku, Katuragi-ku, Tokyo, Japan; 5 Kyoto University Hospital 54 Kawaharacho, Shogoin, Sakyo-ku, Kyoto, Japan

**Keywords:** Cognitive behavioural therapy, Irritable bowel syndrome, Functional gastrointestinal disorder, Group therapy

## Abstract

**Background:**

Irritable bowel syndrome (IBS) is a common disease that affects the quality of life (QOL) and social functioning of sufferers. Visceral anxiety is currently considered a key factor in the onset and exacerbation of IBS, and cognitive-behavioural therapy (CBT) targeting visceral anxiety is thought to be effective. However, access to CBT is limited due to the lack of trained therapists, the substantial time required for therapy and the associated costs. Group CBT (GCBT) may solve some of these problems. We have therefore planned this trial to examine the efficacy of GCBT for IBS.

**Methods:**

The trial is a two-armed, parallel group, open label, stratified block randomized superiority trial. The study group will consist of 112 participants (aged 18–75 years) with IBS (Rome-III or IV criteria). Participants will be randomly allocated 1:1 to (i) the intervention group: ten-week GCBT plus treatment as usual (TAU) or (ii) the control group: waiting list (WL) plus TAU. The co-primary outcomes are the change in IBS severity or disease-specific quality of life from baseline to week 13 which is 1 month after the end of treatment. The efficacy of GCBT for IBS will be examined through mixed-effects repeated-measures analysis.

**Discussion:**

GCBT, if found effective, can address the issues of the shortage of therapists as well as the time required and the costs associated with individual CBT. Clinically, the findings will help make effective CBT programmes accessible to a large number of distressed IBS patients at lower costs. Theoretically, the results will clarify the relationship between IBS and psychological stress and will help elucidate the underlying mechanisms of IBS.

**Trial registration:**

UMIN, CTR-UMIN000031710. Registered on March 13, 2018.

## Background

Irritable bowel syndrome (IBS) is a common functional gastrointestinal disorder characterized by chronic or recurrent abdominal pain and altered bowel habits [1]. Its prevalence among the world population is as high as 10–15%, and IBS is a global health problem [2]. Although IBS is non-fatal, the symptoms affect quality of life (QOL) and social functioning [3] and lead to a health-care burden [4, 5]. Additionally, IBS typically develop in younger adulthood, an important period for education and career development. Thus, the corresponding socioeconomic impact of IBS is considerable [6].

However, there is no gold-standard therapy for IBS, and few options exist for patients who do not respond to available pharmacotherapies. Some psychotherapies, including cognitive behavioural therapy (CBT), hypnotherapy, and stress management therapy, have been proposed for IBS [7, 8]. In particular, CBT for IBS has been studied extensively and a systematic review has estimated the number needed to treat to be approximately 3 [95% CI, 2 to 6] [7]. Also, CBT is recommended in several international guidelines for IBS [9, 10].

Among the several programs reported as CBT for IBS, we focused on the interoceptive exposure-based CBT (CBT-IE) program [11]. CBT-IE was originally developed for panic disorder [12]. Patients with panic disorder are fearful of the sensations that are similar to those that they experience during their panic attacks, and tend to avoid the behaviours and activities that may cause them [13, 14]. These anxieties increase hypervigilance on somatic sensations and are linked to catastrophic thinking [15]. Consequently, even mild somatic sensations produce anxiety and the resultant anxiety intensifies the somatic sensations, thus creating a vicious circle of anxiety and somatic sensations [16]. These interactions between visceral anxiety and somatic sensations are very similar to those observed in patients with IBS [11, 16]. IBS patients show hypervigilance and hypersensitivity to visceral sensations, such as increased intensity of sensations and lowered thresholds for visceral pain [17, 18]. IBS patients often have exaggerated beliefs and anxiety about their symptoms, which make them consciously or unconsciously avoid situations causing the symptoms. Such avoidant behaviours may then increase anxiety, creating a vicious cycle of symptom exacerbation. Thus, CBT-IE approaches that target visceral anxiety using methods similar to those for panic disorder may be suited for IBS [11].

CBT-IE for IBS therefore includes exposure to visceral sensations, in addition to traditional CBT (such as psychoeducation, self-monitoring, cognitive reconstruction, attention training, and in vivo exposure) [11]. In the program, participants are encouraged to experience self-induced visceral sensations (eg, by putting ice on their abdomen) to reduce visceral anxiety. Such bidirectional interaction between somatic sensation and visceral anxiety is consistent with the concept of the brain-gut axis, which is considered to be the mechanism of IBS [19]. Thus, through the use of CBT-IE for IBS, we may be able to provide new insights into the brain-gut axis, a mechanism that plays an important role in the development and maintenance of IBS.

However, access to CBT for patients with IBS is limited due to the paucity of trained therapists, the lengthy time requirements for both the therapists and the patients, and the associated costs [20]. Given the large number of IBS patients and growing health care and social burden, it seems worthwhile to establish the effectiveness of the IBS Group CBT (GCBT). GCBT has been found to be effective in depression, panic disorder and other psychiatric disorders [21], and is attractive since it is cost and time effective [22].

Little is known about the effect of CBT-IE for IBS patients on a group basis. We will conduct a randomized controlled trial (RCT) to examine the efficacy of GCBT for IBS and this study will provide new insights into GCBT for IBS. Our programme aims to break the vicious cycle between anxiety, attention, avoidance behaviour, visceral sensations and disease severity. We will compare GCBT plus treatment as usual (TAU) against TAU alone. Our co-primary outcomes include disease-specific QOL and symptom severity of IBS at week 13 which is 1 month after the end of treatment.

## Methods

The study is a two-arm (allocation ratio 1:1), parallel group, waiting-list controlled, open label, stratified block randomized superiority trial. The reporting of this study protocol follows the Standard Protocol Items: Recommendations for Interventional Trials (SPIRIT) guideline [23] and the recent reporting guideline for interventions [24]. The study will be conducted in accordance with the Japanese Ethical Guidelines for Medical and Health Research Involving Human Subjects (December 22, 2014) and its guidance (revised February 28, 2017).

## Study setting

### Participants

Treatment will take place at the outpatient clinic of the Department of Gastroenterology, Kyoto University Hospital, Japan.

### Eligibility criteria

#### Inclusion criteria

The inclusion criteria are (i) the diagnosis of IBS by a gastroenterologist and medication history for more than 3 months; (ii) age 18–75 years; (iii) an IBS diagnosis according to Rome III or IV criteria* [25]; (iv) moderate or severe symptoms as defined by the IBS Severity Scoring System (IBS-SSS; scores of 175–299 indicate moderate severity, 300–500 indicate severe); (v) the ability to understand Japanese; (vi) the willingness to document bowel symptoms and medication use regularly and to complete the assessments; and (vii) the willingness to attend ten weekly sessions plus one booster session of GCBT.

All subjects will be informed that they should continue to receive usual care from their physicians and that no specific recommendations for changes in medications for IBS will be made by the research team.

Rome III or IV criteria*: In Japan, some patients are still diagnosed and treated according to Rome III, despite the 2016 revision of Roman IV. Thus, we will use both Rome III and IV criteria to include patients representing the IBS population in the clinical practices.

#### Exclusion criteria

The exclusion criteria are (i) patients with past or current psychotherapy**; (ii) outpatients in the psychiatric or psychosomatic medicine departments judged unsuitable for CBT by their doctor; (iii) patients at serious risk of suicide or self-harm as defined by a score of 2 or higher on item 9 of the Patient Health Questionnaire-9 (PHQ-9); (iv) patients who are pregnant; (v) patients with uncontrolled abdominal illness (e.g., active inflammatory bowel disease, liver or pancreatic disease); (vi) patients judged unsuitable for group therapy by the researchers; and (vii) patients who had experience of abdominal operation which had been thought to cause IBS.

Patients with past or current psychotherapy**: Psychotherapy for IBS is rarely practiced in Japan. In this study we aim to examine the benefits and harms of our program among typical patients who will be receiving psychotherapy for the first time. We will therefore exclude patients who have had past or current psychotherapy.

### Interventions

On the first day of trial participation, before randomization, all participants will receive psychoeducation about IBS treatment based on guidelines (e.g., lifestyle and diet, pharmacotherapy) from gastroenterologists [26].

### GCBT

The intervention consists of 10 weekly 90-min sessions with homework tasks and one booster session after 4 weeks. GCBT will be delivered face-to-face in groups of up to four participants and one or two therapists.

We prepared a manual translated into Japanese according to the Interoceptive Management for IBS programme by Craske et al. [11]. The original programme was designed for individual sessions, but with the permission of the original authors, we have modified the manual to accommodate group therapy and have prepared handouts for participants. The contents of the programme include the following:

- Explanation of a psychological model of IBS. The effect of anxiety on gastrointestinal functioning and the role of conditioning in IBS. Patients record their own IBS-related behaviours (session 1).
- The role of awareness of IBS-related stimuli. Attentional control skills to learn how to shift attention away from rather than perseverate upon unpleasant visceral sensations (session 2).
- Explanation of the role of negative thoughts in exacerbating IBS (session 3).
- Explanation of how IBS-related avoidance and control behaviours maintain the fear and awareness of IBS symptoms (session 4).
- Interoceptive exposure (e.g., place ice on the stomach to cause abdominal symptoms similar to IBS symptoms) to reduce fear of the sensations (sessions 4 & 5).
- Exposure to feared/avoided situations in which IBS sensations are expected (sessions 4 & 5).
  1. Reduction or removal of behaviours that serve to control symptoms, such as repeated toilet visits, avoidance of certain foods, and taking medications.
  2. Exposure to symptoms by engaging in activities that provoke symptoms, such as eating certain foods, physical activity, and stressful situations, attending a meeting when experiencing abdominal pain or riding the bus with fear of losing bowel control.
- Education for how to handle relapse (session 10).

Two therapists will conduct the GCBT treatment; one gastroenterologist with 13 years of experience in the treatment of IBS; and one clinical psychologist with 10 years of clinical experience. Both therapists had had little experience with GCBT with interoceptive exposure for IBS, and they have completed supervised GCBT training with a clinical psychologist who has extensive experiences with CBT for anxiety disorders in general and with CBT for IBS. All sessions will be performed using the treatment manuals that provide detailed guidance to standardize intervention during the study: the two therapists will also continue to receive supervision as needed through the current trial.

The participants allocated to the intervention group will start the GCBT immediately after randomization. They will concomitantly receive TAU from their general practitioner or gastroenterologist during the GCBT intervention.

#### Waiting-list (delayed start) control group

The control condition of this study is a waiting list. During the waiting period, the participants will receive TAU and will also be asked keep an IBS diary for self-monitoring [27]. The participants in the control condition will receive GCBT after week 13.

#### Treatment as usual (TAU)

TAU is defined as the continuation of current medications prescribed by a general practitioner or gastroenterologist. All participants will continue TAU during the study period. We will collect and record information about any changes in IBS treatments/management during the study.

#### Other treatments

We will ask all participants not to change their TAU as much as possible during the study period. We will record any change in medications for IBS. Other psychotherapies for IBS are prohibited during the study period. There is no restriction on the treatments for comorbidities.

#### Criteria for discontinuing the allocated interventions

The allocated intervention will be discontinued if any of the following criteria are met:

- If the participant wishes to withdraw from the intervention for any reason
- If the participant cannot continue the intervention for any reason (e.g., death, exacerbation of comorbidities, adverse events, etc.)
- If the study itself is discontinued.
- If the steering committee determines that it is appropriate to stop the intervention.

We will record the reason and date of discontinuation. We will follow up and assess the participants after the discontinuation of the allocated interventions as long as they do not withdraw their consent to be evaluated.

### Outcomes

As there is no standard outcome measure for IBS [28], we will use two clinically important measures as the co-primary outcomes of this study, namely, the severity of symptoms of IBS and disease-specific QOL due to IBS [29]. When one variable cannot fully capture the range of therapeutic efficacy, it is sometimes necessary to use several variables [30, 31]. We will consider the intervention efficacious if at least one of the co-primary outcomes shows statistically significant superiority at the pre-designated alpha-level controlling for multiple outcomes [30].

### Primary outcomes

The two co-primary outcomes are changes in IBS-SSS and/or IBS-QOL scores from baseline to week 13.

#### The irritable bowel syndrome severity scoring system (IBS-SSS)

The IBS-SSS is a 5-item self-administered questionnaire. The IBS-SSS relates to individual domains for the severity of abdominal pain, the duration of abdominal pain, bloating, satisfaction with bowel habits, and QOL. The IBS-SSS total score ranges from 0 to 500: < 75 normal, 75–174 mild IBS, 175–299 moderate IBS, 300–500 severe IBS. According to the scale developers, a 50-point or greater change on this scale is considered clinically significant [32, 33].

#### The irritable bowel syndrome quality of life (IBS-QOL)

The IBS-QOL is a 34-item, 8-subscale self-administered questionnaire. The IBS-QOL relates to individual domains for dysphoria, interference with activity, body image, health worry, food avoidance, social reaction, sexual relationships. The total score ranges from 0 to 100, with lower scores reflecting poorer QOL [34, 35]. Evidence shows that a range from minimal to optimal response may be in the 10–14 point range [36].

### Secondary outcomes

Our secondary outcomes include (a) the Gastrointestinal Symptom Rating Scale (GSRS: a clinical rating scale for gastrointestinal symptoms with 15 items, 7 subscales) [37, 38]; (b) the Irritable Bowel Syndrome-Global Improvement Scale (IBS-GIS: measures patient-perceived severity of symptoms with a 7-point Likert-type scale ranging from 0 [no discomfort] to 6 [very severe discomfort]) [39]; (c) the Patient Health Questionnaire-9 (PHQ-9: 9 items, 4 subscales, scale 0–27; lower scores indicate lower symptom severity of depression) [40]; (d) the Generalized Anxiety Disorder-7 (GAD-7: 7 items, 4 subscales, scale 0–21; lower scores indicate lower generalized anxiety) [41]; (e) EuroQol-5 Dimension-5Level (EQ-5D-5 L: health status is assessed with 5 items, 5 subscales) [42] and a visual analogue scale (VAS); (f) the Composite Primary Reduction Score (CPRS: 4 items, 5 subscales, lower scores indicate lower abdominal symptoms).

We will also collect data on medication use and health services used. Along with baseline data, all outcome data will be sought by the Internet or a postal questionnaire at 4, 9, 13, 27 weeks, and where these methods fail, data for the main outcome measure will be sought by telephone. We will also seek information associated with the homework completion rate and adverse events (AEs).

### Participant timeline

**Fig. 1:**
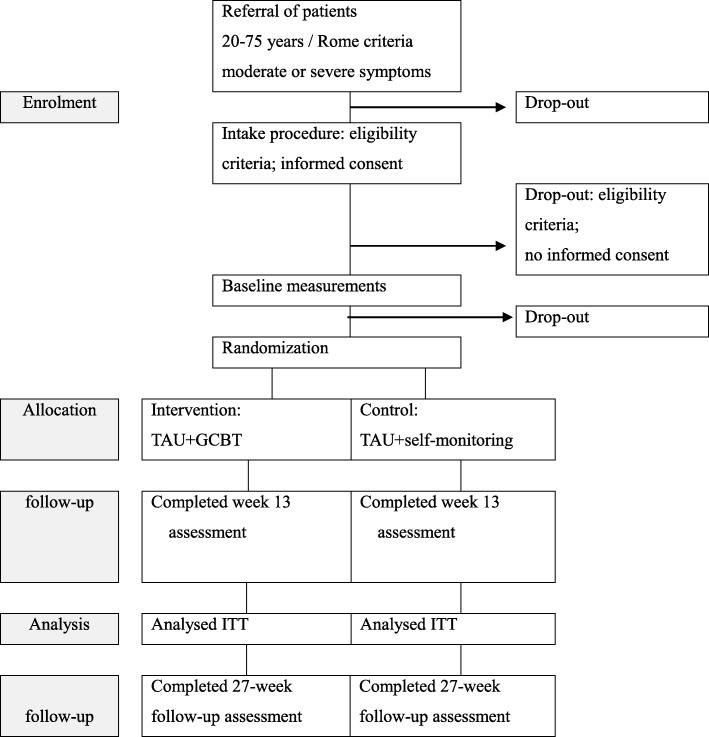
The participant timeline

Figure 1 shows the participant timeline, and Table. 1 shows the enrolment, intervention, and assessment schedule.

**Table 1:**
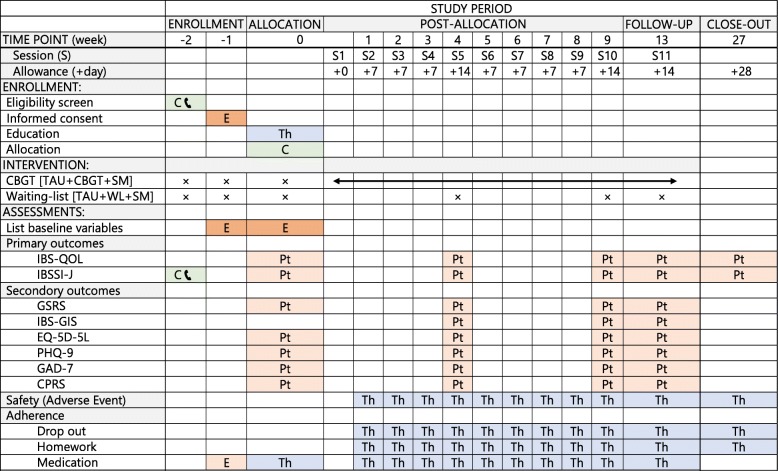
The enrolment, intervention, and assessment schedule

Note: Evaluation is performed by the person indicated by each colored cells

*Abbreviations*: *C* Coordinator, *C*  Coordinator calls patient, *E* Evaluator, *Pt* Patient, *Th* Therapist

### Sample size

The sample size is based on the two co-primary outcomes of IBS-SSS and IBS-QOL. To adjust for multiplicity conservatively, we used the Bonferroni method and set the significance level at 2.5% (α level: 0.025). The correlation coefficient between the absolute scores of IBS-SS and IBS-QOL has been reported to be 0.36 [35]. With power set at 80% and α set at 0.025, for two co-primary outcomes correlating at 0.40, a sample size of 55 subjects per group is required to obtain sufficient power to detect a moderate effect size of 0.5 [43] between the groups. Because each randomization will take place for a group of 8 (4 being assigned to the intervention and 4 to the control), a minimum of 14 groups is required to reach this number, and the total sample size is therefore 8 × 14 = 112. With the mixed-effect models for repeated measures, we can obtain the same power with 30–50% fewer participants than with the ordinary t-test or analysis of covariance [44]. In this trial, less than 30% dropout is expected. We therefore set the final target sample size, including drop-outs, as 112 patients in total, or 56 patients in each group. The power calculations were conducted with SAS PROC POWER by an independent statistician (TS). The appendix shows the SAS program.

### Recruitment

Participants will be recruited from primary and secondary care in Kyoto and surrounding prefectures and through the Internet. We are planning to recruit the participants within 2 years. Should there be any difficulty recruiting the planned sample size, we will cooperate with hospitals and clinics outside of Kyoto city.

### Assignment of interventions

#### Allocation

##### Sequence generation

The independent statistician (TS) will generate the random allocation sequence using SAS 9.4. We will use permuted block randomization with a block size of 4 for stratified randomization by baseline severity.

##### Randomization and allocation concealment

Up to eight participants will attend the initial IBS psychoeducation session. A researcher who is not involved in participant recruitment, assessment or treatment will receive the allocation sequence in a sealed envelope from the principal investigator and will receive the data about the participants’ IBS-SSS scores from the gastroenterologist conducting the baseline assessment. The group will be split by the median score into the more severe and the less severe halves, and the independent researcher will then allocate each participant to either GCBT or WL according to the corresponding sequence and will notify the treatment team of the allocation results.

##### Blinding

Blinding is notoriously difficult in research on psychotherapy. In this research, neither participants nor researchers will be blinded to the intervention that each participant is receiving. Furthermore, the primary and secondary outcome assessments are not blinded because these are self-administered PROM measures.

The data management team will prepare the datasets in which all components are denoted only by a letter. The statistician will be blinded to the allocation during the statistical analyses until the statistical analysis report is finalized.

### Data collection

#### Data collection methods

Outcome data and questionnaires will be completed at baseline, the midpoint (week 4), the end of the treatment (week 9), 4 weeks after the treatment (week 13), and during the follow-up (week 27) by all participants. Participants will receive a reminder email or a telephone call to complete the questionnaires 1 week prior to the deadline.

#### Plans to promote participant retention and complete follow-up

To avoid bias due to dropout, we will carefully follow the participants in both groups. We will collect the outcome data even if participants drop out from the allocated intervention, as long as they provide consent for the assessments.

Reminder emails will be sent within 24 and 48 h if participants do not fill out the questionnaire on the scheduled due date via Research Electronic Data Capture (REDcap) (https://www.project-redcap.org/). Participants who select paper questionnaires will be reminded by phone if there is no reply by the scheduled date. To reduce the burden of participating in the research, participants will receive a small incentive (1000 yen) for each of the five evaluations at baseline and 4, 9, 13, and 27 weeks. If the questionnaires are not completed within 1 week, the researcher will ask the participant if they can collect the data over the telephone.

#### Data management

The participants will fill in the questionnaires through the Internet on REDcap. The researchers will enter the data into REDcap regarding eligibility criteria, adverse events, changes in medication and treatments, and homework adherence, by the double data entry method. If there is a missing value in the data, the researcher will make inquiries by telephone. We will conduct central monitoring of the data collected at the data centre via REDcap.

### Statistical methods

All analyses will be performed according to the intention-to-treat principle; all participants will be included in their randomized groups whether or not they have received their allocated treatment.

The mean difference in change scores on the IBS-SSS and the IBS-QOL will be estimated by the mixed-effects model for repeated measures (MMRM) [45]. In this model, the scale scores at weeks 4, 9 and 13 are the dependent variables. The allocation group (intervention = 0 vs control = 1), measurement point (middle = 1, post = 2, follow = 3) and the interaction term of both factors are fixed effects. The measurement point is treated as a categorical variable. The baseline measurement is used as the covariate. A participant is taken as the random effect. The covariance matrix describes the correlation between observations at different measurement points [45]. The Hochberg method will be applied for adjustment of multiplicity, and the threshold for statistical significance will be set at *p* < 0.05 (two-sided).

Secondary outcomes, including GSRS, IBS-GIS, PHQ-9, GAD-7, EQ-5D-5 L and CPRS, are important to measure the wider IBS effects and will be similarly analysed (as appropriate for continuous or dichotomous outcomes). These analyses will be exploratory in nature to complement the primary analyses; therefore, we will not adjust for multiple testing.

In this trial, participants who attend at least 1–5 sessions will be defined as a ‘Completer’, and we will conduct an analysis of covariance (ANCOVA) using the completers’ data and check the robustness of the result by MMRM.

The follow-up assessments at week 27 will be descriptively compared with the assessments at week 13, as the randomized comparison between the two arms no longer holds after the control participants have received the GCBT.

### Monitoring

#### Interim analysis

No interim analysis is planned.

#### Harms

All serious adverse events regardless of the intervention, defined as death, life-threatening events, hospitalization (initial or prolonged), disability or permanent damage, and congenital anomaly/birth defect, will be handled according to the procedures set out by Kyoto University Hospital and reported to the ethics committee.

Patients with IBS may present with elevated depression, and the risk for suicide attempts cannot be ruled out. We will therefore monitor patients through the PHQ-9.

#### Auditing

Since the intervention in this research can be classified as a “minimally invasive intervention”, formal audits will not be conducted.

## Ethics and dissemination

### Research ethics approval

This study protocol is based on protocol version 1.2 by the ethics committee of Kyoto University Graduate School of Medicine (C1360) and the National Center of Neurology and Psychiatry (A2018–019).

### Protocol amendments

Revision of the protocol will be decided by the steering committee. Any amendments to the protocol will be submitted to the ethics committee of Kyoto University Graduate School of Medicine for approval. After this approval, the amended protocol will be submitted to the ethics committee of the other participating institutions and reported to the participants as necessary.

### Consent or assent

We will implement a two-step consent procedure. First, candidates for participation will be introduced to the secretariat. These candidates will undergo the eligibility criteria check and be given an explanation about the trial via telephone. The researcher will obtain oral consent from candidates who satisfy the eligibility criteria. Second, participants who have consented orally will visit Kyoto University Hospital and receive information through a face-to-face interview. The researchers will obtain the fully informed written consent to participate in the trial of GCBT for IBS.

Because the participants in this study are over 18 years of age and the intervention is minimally invasive, consent from representatives is not required.

### Confidentiality

Participants will receive an identification number at the time they agree to participate. This identification number will be used for all data registration. The correspondence table between the identification number and the patient’s personal information will be documented (not digitised) and kept in a locked drawer. Consent forms, patient background information, and questionnaire data will be stored securely in the web-based, password-protected database at Kyoto University Hospital. Audio recordings of the sessions will be stored on the hard disk, protected by a separate password; the hard disk will be securely stored in a locked laboratory room along with the paper-based questionnaires.

Once the trial is completed, the raw data will be kept in a locked drawer at the Department of Health Promotion and Human Behaviour, Kyoto University Graduate School of Medicine/School of Public Health for 10 years after the first survey results are published.

### Access to data

All members of the steering committee and the statistician will have full access to the final trial dataset. The de-identified, anonymized dataset will be uploaded to the UMIN-ICDR website (http://www.umin.ac.jp/icdr/index-j.html), and researchers approved by the steering committee will be able to have access to the dataset.

### Ancillary and post-trial care

All participants can receive appropriate treatment immediately, whenever any such treatment is necessary, within the framework of TAU provided to them during the study. Medical expenses for such treatment will be covered by each participant’s health insurance, and no monetary compensation will be provided.

After the trial, TAU will be offered. There is no restriction on changing medications and receiving new psychotherapy after trial participation.

### Dissemination policy

After approval by the ethics committee of Kyoto University, this research was registered on UMIN-CTR (http://www.umin.ac.jp/ctr/index-j.htm), and the protocol paper will be published in an English language journal.

*Research* results will be made available to medical professionals and the public through academic journal publications and at academic conferences. The authors of the paper and the conference presenters will be decided according to the Uniform Requirements for Manuscripts Submitted to Biomedical Journals of the International Committee of Medical Journal Editors [46].

## Discussion

We have described the RCT protocol of GCBT with interoceptive exposure for IBS patients. The primary objective of this study is to evaluate the effectiveness of GCBT for IBS and contribute to the provision of a scientific basis for non-pharmacotherapy to treat refractory IBS. The study also has several secondary objectives, such as changes in anxiety and depression.

Group therapies are cost and time effective. In addition, group therapy has the advantage of allowing participants to interact with others with similar problems and are expected to have mutual support capabilities through sharing experiences and coping models in difficult situations among themselves [47, 48]. However, only a few randomized controlled trials (RCTs) of GCBT for IBS have been reported, and the IBS diagnostic criteria, control groups, and outcome measures used in each study were different. Moreover, two of the studies were small [49, 50], and one of them reported inappropriate randomization [49]. About the effectiveness of GCBT for IBS, their findings have been mixed [49–51]. Two RCTs confirmed the effect of GCBT for IBS; one study enrolled 47 IBS participants (diagnostic criteria by researchers) and found that GCBT was superior to the waiting-list group in alleviating abdominal complaints at 3 months [49]; another study randomized 23 IBS patients (Rome criteria) and found that GCBT produced greater reduction in gastrointestinal symptoms at 3 months than the home-based symptom monitoring with weekly telephone contact [50]. In contrast, a third study concluded that GCBT is not superior to an attention placebo control condition. The last study enrolled 210 IBS patients (Rome II criteria) and studied the effects of GCBT versus psychoeducational support versus intensive symptom and daily stress monitoring, on patients’ GI symptom for 3 months [51]: Both GCBT and psychoeducational support were significantly more effective than intensive symptom monitoring, but GCBT and psychoeducational support group did not differ.

The salient features of our CBT program include: (i) addition of interoceptive exposure; (ii) removal of relaxation training; and (iii) use of the treatment manual. CBT-IE was developed using CBT for panic disorder as a model, aiming to correct excessive visceral anxiety and avoidance behaviour through cognitive restructuring and repeated exposures to somatic sensations [11]. Many psychotherapies for IBS include stress management with relaxation training. However, recent studies showed that reducing anxiety and avoidance behavior through interoceptive exposure resulted in higher quality of life than reducing stress responsiveness to daily life through relaxation training. We therefore dropped relaxation training from CBT-IE for IBS [11].

Also, few therapists, either clinical psychologist, psychiatrist or gastroenterologist, have experience with CBT for physical diseases such as IBS in Japan. The therapists of this RCT have therefore had little experience with CBT for physical diseases and will conduct therapies after brief but intensive training based on the manual. We consider that GCBT for IBS can be more widely practiced, if it can be effectively conducted by less skilled therapists using the manual.

### Strengths

Our study has several strengths. First, to our knowledge, this study will be the first RCT of Group CBT-IE for IBS in the world and it may provide new insights about the effectiveness of GCBT for IBS. Second, the use of the manual-based GCBT, if shown effective, is expected to solve the issue of the shortage of CBT therapists and increase the cost effectiveness. Finally, participants are recruited from multiple sources representing the primary care and the secondary/tertiary centers in Kyoto, and the results can be expected to have generalizability.

### Limitation

There are some limitations to this study. First, this study is an open-label randomized controlled trial, in which participants, personnel and outcome assessor are unblind and only the data analyst is blinded. Unblinding of participants and personnel can result in performance bias, and results may be biased in favour of the intervention group [52]. Moreover, there is no standard indicator (eg, biomarker etc.) that can be used as an objective primary endpoint in IBS. Thus, the primary outcome of this study is IBS disease severity and disease-related quality of life, which are measured by patient reported outcomes (PROs). Although the importance of PROs has been recognized, the absence of blinding of outcome assessment can result in detection bias [53]. To minimize this bias, we will use only scales that have been established with confidence and validity.

## Data Availability

After publication of the primary findings, the de-identified and completely anonymised individual participant-level dataset will be posted on the UMIN-ICDR website (http://www.umin.ac.jp/icdr/index-j.html) so that it can be accessed by qualified researchers.

## Abbreviations

ANCOVA: Analysis of covariance
CBT: Cognitive behavioural therapy
EQ-5D-5 L: EuroQol - 5 dimension - 5Level
GAD-7: Generalized anxiety disorder-7
GCBT: Group cognitive behavioural therapy
IBS: Irritable bowel syndrome
IBS-GIS: The Irritable Bowel Syndrome-Global Improvement Scale
IBS-QOL: The Irritable Bowel Syndrome Quality of Life
IBS-SSS: The Irritable Bowel Syndrome Severity Scoring System
MMRM: Mixed-effects model for repeated measures
PHQ-9: The Patient Health Questionnaire-9
QOL: Quality of life
RCT: Randomized controlled trial
REDcap: Research Electronic Data Capture
TAU: Treatment as usual
VAS: Visual analogue scale
WL: Waiting List
